# Rare Case of Tension Pneumocephalus in Thoracic Trauma

**DOI:** 10.7759/cureus.16136

**Published:** 2021-07-03

**Authors:** Oana M Stroie, Kyle V Keinath, Christopher M Knaus

**Affiliations:** 1 Diagnostic Radiology, Uniformed Services University of the Health Sciences, Bethesda, USA; 2 Diagnostic Radiology, Walter Reed National Military Medical Center, Bethesda, USA

**Keywords:** tension pneumocephalus, thoracic trauma, pneumocephalus, mount fuji sign, neurosurgery

## Abstract

Tension pneumocephalus is the presence of air within the cranial vault compressing the ventricles and the brain parenchyma. High altitudes can exacerbate this problem, especially when a dural defect exists and air is forced into the cranial cavity with no way to escape. This case demonstrates a rare presentation of thoracic trauma causing tension pneumocephalus due to emergent air evacuation.

## Introduction

Pneumocephalus is the presence of air within the cranial vault. It is most often associated with craniofacial trauma, especially injuries involving the basilar skull. Other causes include cranial surgery, nasopharyngeal tumor invasion, infection, and epidural or spinal anesthesia [1,2]. There is a very limited number of reported cases involving thoracic and lumbar trauma, as in ours. Clinically, pneumocephalus may be asymptomatic. However, depressed mental status, headache, confusion, nausea, vomiting, seizures, dizziness, Cushing response, restlessness, focal neurological deficits, and cardiac arrest have been described and are more associated with tension versus nontension pneumocephalus [1,3,4].

Two mechanisms have been proposed to explain pneumocephalus, namely, “the inverted soda bottle mechanism” and “the ball valve mechanism.” In the first, leakage of cerebrospinal fluid (CSF) creates negative intracranial pressure causing a vacuum-like effect with additional accumulation of air within the cranial cavity until the pressure gradient is fixed or stable. The second is based on the presence of a one-way valve at the site of the leptomeningeal tear, where air is forced into the cranial cavity with no way to escape [5].

## Case presentation

**Figure 1:**
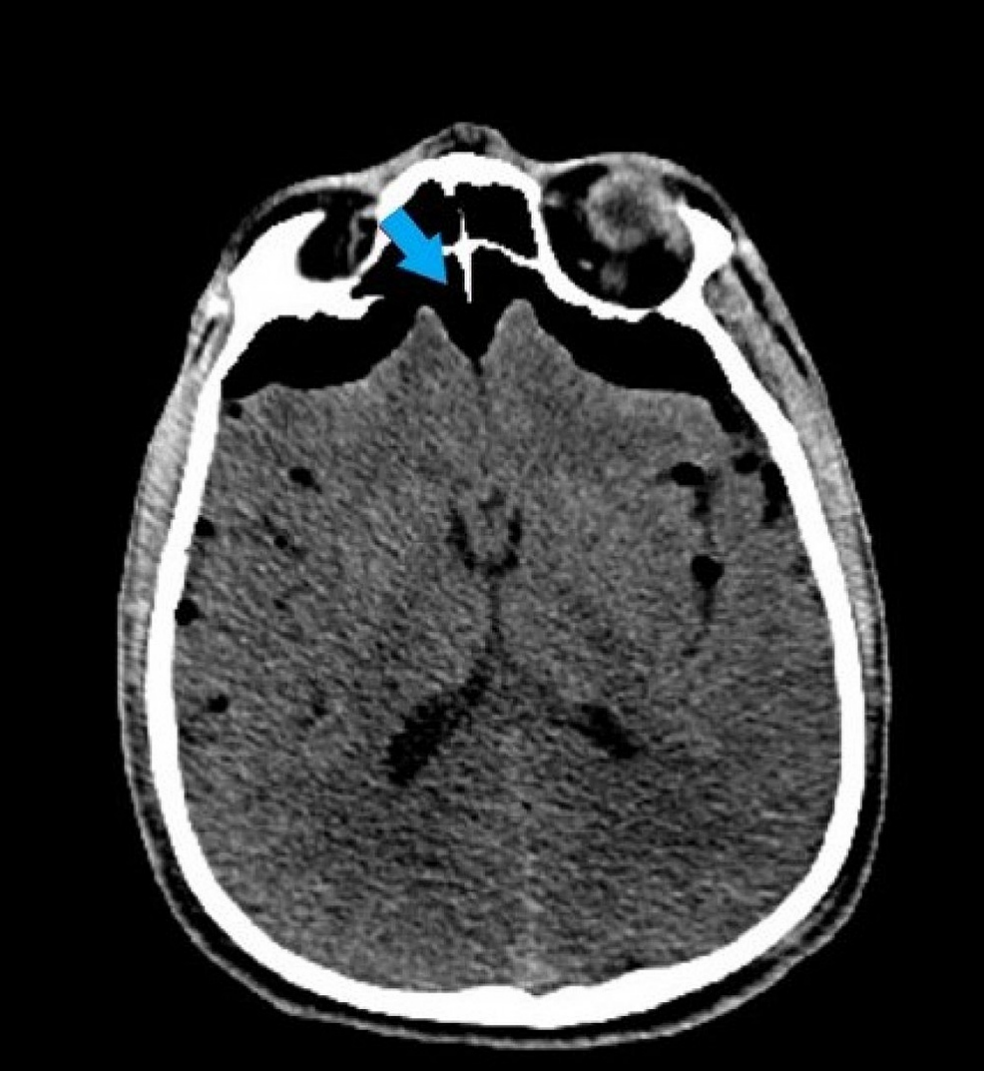
Noncontrast head CT through the frontal sinuses demonstrates the “Mount Fuji sign” of tension pneumocephalus (blue arrow). CT: computed tomography

**Figure 2:**
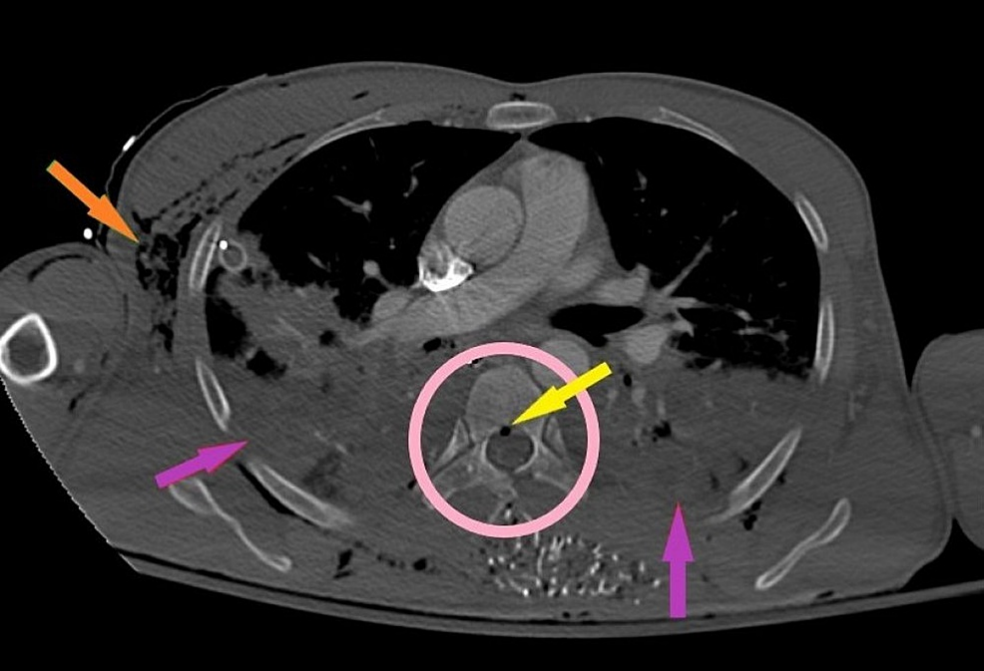
Contrast-enhanced CT with an axial bone window of the chest. The orange arrow points to subcutaneous emphysema. The purple arrow points to bilateral hemorrhagic pleural effusions. The pink circle highlights the right costovertebral junction, right pedicle, and left lamina of T6 that is completely shattered. The yellow arrow points to hemorrhagic material in the spinal cord with punctate foci of gas. CT: computed tomography

A 45-year-old man was air evacuated with a gunshot wound to the chest. His condition worsened during transport. On arrival, CT imaging revealed multiple penetrating chest traumas and tension pneumocephalus (Figures 1, 2). The patient died soon afterward.

## Discussion

The case is poignant especially for military medical providers operating in austere environments where patient transportation can often involve aircraft flights. On experiencing reduced atmospheric pressure, the volume of air within the cranium of a tension pneumocephalus patient will expand, as defined by PV = nRT (the ideal gas law equation states that pressure multiplied by volume equals the number of gas moles multiplied by the universal gas constant and temperature). This expansion can cause an increased mass effect on the brain. In our case, given the physiology at initial presentation, we believe that an expanded tension pneumocephalus caused early brain herniation with pressure upon the brain stem causing central respiratory compromise and death.

In our case, thoracic penetration with associated spinal fracture allowed air entry into the spinal canal by the “ball valve mechanism” with direct air entry into the subarachnoid space creating an expanding tension pneumocephalus that was further exacerbated by changes in elevation during emergent air transport.

Tension pneumocephalus is a neurosurgical emergency and early identification is key to treatment. The entry of air can often create an early “peaking sign” with the compression of the frontal lobes on CT head imaging. As the entry of air surpasses the surface tension between CSF and the frontal lobes, it leads to a collapse of the frontal lobes and widening of the interhemispheric space creating the classic “Mount Fuji sign” that is characteristic of tension pneumocephalus [6]. When tension pneumocephalus is detected via imaging, immediate neurosurgery treatment is needed in the forms of needle aspiration, drilling of burr holes, craniotomy, ventriculostomy, and closure of the dural defect [5,6].

## Conclusions

Tension pneumocephalus is a very rare complication of penetrating spine trauma that can be exacerbated by flight evacuation, and therefore, needs to be considered when transporting patients. The “Mount Fuji sign” is a continuation of the early “peaking sign” on head imaging. It is a characteristic finding of tension pneumocephalus and indicates that the gas is exerting a mass-like effect that exceeds the surface tension of CSF leading to a collapse of the frontal lobes and widening of the interhemispheric space. Tension pneumocephalus is a neurosurgical emergency and can be a result of trauma, infections, erosive pathology of the sinuses, and iatrogenic from neurosurgery or intrathecal anesthesia.
